# Re-evaluation of a Neonatal Mouse Model of Infection With Enterotoxigenic *Escherichia coli*

**DOI:** 10.3389/fmicb.2021.651488

**Published:** 2021-03-18

**Authors:** Carla J. Carroll, Dianna M. Hocking, Kristy I. Azzopardi, Judyta Praszkier, Vicki Bennett-Wood, Kaylani Almeida, Danielle J. Ingle, Sarah L. Baines, Marija Tauschek, Roy M. Robins-Browne

**Affiliations:** ^1^Department of Microbiology and Immunology, The University of Melbourne at the Peter Doherty Institute for Infection and Immunity, Parkville, VIC, Australia; ^2^Murdoch Children’s Research Institute, Royal Children’s Hospital, Parkville, VIC, Australia

**Keywords:** enterotoxigenic *E. coli*, animal model, F41 pili, heat-stable enterotoxin, molecular Koch’s postulates

## Abstract

Enterotoxigenic *E. coli* (ETEC) is a common cause of diarrhea in children in low- and middle-income countries, and in travelers to these countries. ETEC is also an important cause of morbidity and premature mortality in piglets, calves, goat kids and lambs. The major virulence determinants of ETEC are enterotoxins and colonization factors, which enable the pathogen to colonize the small intestine and deliver enterotoxins, such as the heat-stable enterotoxins, STp and STh, to epithelial cells. Because most ETEC strains are host-specific, there are few convenient animal models to investigate the pathogenesis of ETEC infections or to evaluate specific anti-ETEC interventions, such as drugs and vaccines. An exception is ETEC strains bearing F41 pili, which mediate intestinal colonization of various young animals, including neonatal mice, to cause disease and in some cases death. In this study, we used the archetypal F41-producing bovine ETEC strain, B41 (O101:NM; K99, F41, STp) to validate and further explore the contribution of F41 and STp to bacterial virulence. By using targeted gene deletion and trans-complementation studies, augmented by whole genome sequencing, and *in vitro* and animal studies of virulence, we established that F41 mediates colonization of the mouse intestine and is essential for bacterial virulence. In addition, we showed for the first time that STp is as important as F41 for virulence. Together, these findings validate the use of neonatal mice to study the pathogenesis of F41-bearing ETEC and to investigate possible specific anti-ETEC interventions including vaccines that target heat-stable enterotoxins.

## Introduction

Enterotoxigenic *Escherichia coli* (ETEC) is a leading cause of diarrhea in children in low- to middle-income countries and in travelers of all ages to these countries (Qadri et al., 2005; Croxen et al., 2013; Kotloff et al., 2013). ETEC is also an important cause of diarrhea in young farm animals, in particular piglets, calves, lambs, and goat kids (Dubreuil et al., 2016).

ETEC are characterized by two categories of virulence determinant: secreted enterotoxins and surface-expressed colonization factors (Croxen et al., 2013). The enterotoxins are further divided into two broad groups, known as heat-stable toxin (ST) and heat-labile toxin (LT) (Croxen et al., 2013). The commonest variety of ST, STa, is highly prevalent in human varieties of ETEC, as well as in strains obtained from newborn domestic animals. It is a weakly antigenic protein comprising 18 (STp) or 19 (STh) amino acids, respectively (Fleckenstein et al., 2010). Although STh (also known as STIb) is more frequent in ETEC obtained from humans than STp (also known as STIa), these two toxins are structurally similar, have an identical mechanism of action and are antigenically related. LT, on the other hand, is a large (ca. 86 kDa) protein containing one A-subunit and five identical B-subunits. This toxin resembles cholera toxin structurally, functionally, and antigenically (Kunkel and Robertson, 1979).

The colonization factors, which are the other essential virulence determinant of ETEC, mediate bacterial adhesion to the small intestinal mucosa where the enterotoxins mainly act. These adhesins vary considerably in terms of their amino acid composition, structure, morphology and antigenic relatedness (Gaastra and Svennerholm, 1996; Croxen et al., 2013). Although the receptors for most of these colonization factors are not known, these receptors are generally host specific, meaning that there is negligible overlap between the host range of individual ETEC strains, particularly between those found in humans and those from domestic animals.

The number of different colonization factors of ETEC obtained from symptomatic humans exceeds 20, whereas those obtained from diarrheic piglets, calves, goat kids or lambs are limited to around six, including F4 (also known as K88), F5 (K99), F6 (987P), F17, F18, and F41. Interestingly, some of these colonization factors overlap in their host range. The least specific of these factors is F41, a fimbrial adhesin of bovine ETEC that is also able to mediate intestinal colonization of lambs, goat kids and piglets (Dubreuil et al., 2016; Luppi, 2017). The basis of F41’s versatility is unknown but could be due to its ability to bind to different receptors (Lindahl and Wadström, 1986) or to the same receptor in different hosts.

In addition to infecting various natural hosts, ETEC strain B41, the archetypal bovine ETEC strain in which F41 was first identified, can also infect suckling mice to cause an infection that is often fatal (Duchet-Suchaux, 1980; Bertin, 1983). This makes *E. coli* B41 a convenient tool with which to investigate the pathogenesis of ETEC infections in a small animal model, as well as a means to evaluate early phase, anti-ETEC therapies and vaccines. Another unusual feature of *E. coli* B41, is that, apart from the chromosomally encoded F41 adhesin, it also carries a plasmid for K99. While K99 is likely to enhance the pathogenicity of B41 for calves, lambs and piglets, it does not play a major role in B41’s virulence for neonatal mice (Bertin, 1985; Duchet-Suchaux, 1988).

Previous studies have provided strong circumstantial, but incomplete, evidence that F41 is an essential virulence determinant of *E. coli* B41, and none has investigated the role of its STp enterotoxin in the mouse model. The aim of the current study was to determine the contributions of F41 and STp to the virulence of *E. coli* B41 in a neonatal mouse model of ETEC infection.

## Materials and Methods

### Bacterial Strains, Plasmids, Primers, and Media

Bacterial strains and plasmids used in this study are listed in Table 1. PCR primers were purchased from GeneWorks and are shown in Supplementary Table 1.

**TABLE 1:** Bacterial strains and plasmids used in this study.

Strain or plasmid	Relevant characteristics	References/source
***E. coli* strains**		
B41	Streptomycin-resistant derivative of bovine ETEC strain O101:NM; F41, K99, STa, Sm^*R*^	Smith and Halls, 1967
B41ΔF41	B41 *f41A*:*aph(3′)-IIa*, Km^*R*^	This study
B41ΔF41 (F41)	B41ΔF41 (pACYC184:*f41A*), Km^*R*^, Cm^*R*^	This study
B41ΔSTp	B41 *estA*:*aph(3′)-IIa*, Km^*R*^	This study
HS	Non-pathogenic *E. coli* of human origin	Levine et al., 1978
MC4100	F^–^*araD*139 (*argF*-*lac*) *lacU*169 *rpsL*150 *relA*1 *flbB*5301 *deoC*1 *ptsF*25 *rbsR thiA*	Casadaban, 1976
TOP10	F^–^ *mcrA* Δ(*mrr*^–^, *hsdRMS*^–^, *mcrBC*) φ80*lacZ*ΔM15 Δ*lacX*74 *nupG recA*1 *araD*139 Δ(*ara-leu*)7697 *galE*15 *galK*16 *rpsL*(Str^*R*^) *endA*1 λ^–^	Invitrogen
**Plasmids**		
pACYC184	Medium-copy number vector, Cm^*R*^, Tc^*R*^	Chang and Cohen, 1978
pACYC184:*f41A*	pACYC184 containing *f41A*, the gene for the	This study
	29 kDa major subunit of the F41 pilus, Cm^*R*^	
pCR2.1-TOPO	High-copy number vector, Ap^*R*^, Km^*R*^	Invitrogen
pGEM-T Easy	High-copy number vector, Ap^*R*^	Promega
pKD4	Vector containing Km^*R*^ gene, Ap^*R*^, Km^*R*^,	Datsenko and Wanner, 2000
pKD46	Low-copy number vector, P_*BAD*_-λred, Ap^*R*^	Datsenko and Wanner, 2000
pXen-13	pSK *luxCDABE*, Ap^*R*^	Xenogen

*Ap^R^, ampicillin resistance; Cm^R^, chloramphenicol resistance; Km^R^, kanamycin resistance; Sm^R^, streptomycin resistance; Tc^R^, tetracycline resistance.*

Except where indicated, bacteria were grown in Luria-Bertani broth (LB) or on Luria agar (LA) at 37°C. When required, antibiotics were used at the following concentrations: ampicillin 100 μg/ml, chloramphenicol 10 μg/ml, kanamycin 40 μg/ml, streptomycin 50 μg/ml. Also used were Trypticase soy agar (TSA), SOB and SOC media (Hanahan, 1983). Minimal casein (Minca) media were prepared as described (Guinée et al., 1976) and used to grow bacteria when expression of F41 pili was desired (de Graaf and Roorda, 1982). To enhance production of enterotoxin, bacteria were grown in casamino and yeast extract (CAYE) medium (Evans et al., 1973). All chemical reagents were purchased from Ajax Chemicals, Sigma-Aldrich or BDH Laboratory Supplies unless otherwise specified.

### DNA Manipulation

Restriction enzyme digestions were performed using enzymes and buffers from New England BioLabs (NEB) according to the manufacturer’s instructions. DNA sequencing of PCR amplicons was performed using the BigDye terminator (v3.1) cycle sequencing kit (Applied Biosystems) in accordance with the manufacturer’s instructions. Sequencing reactions were completed in a GeneAmp PCR system 9700 thermal cycler (Applied Biosystems), and the results were analyzed using Sequencher (Gene Codes). PCR amplifications were performed using GoTaq Green Master Mix (Promega), or Phusion Flash High-Fidelity PCR Master Mix (Finnzymes). To construct the plasmids used in this study, we first cloned the various PCR fragments into pCR2.1-TOPO (Invitrogen/Life Technologies) or pGEM-T Easy (Promega). Following sequence verification, we cloned the various inserts from the pCR2.1-TOPO or pGEM-T Easy derivatives into the appropriate vectors (Table 1).

### Construction of F41 and STp Knockout Mutants of *E. coli* B41 and Their Bioluminescent Derivatives

The λ Red recombinase system (Datsenko and Wanner, 2000) was used to construct a knockout mutation in *f41A*, the gene for the 29-kDa, major structural subunit of the F41 pilus of *E. coli* B41 (Fidock et al., 1989). First, the Phusion high-fidelity DNA polymerase, the primer pairs, F41.F1/F41.R1, and plasmid, pKD4, were used in a PCR reaction to generate a DNA fragment that contained the kanamycin-resistance gene (*aph(3′)-IIa*) flanked by ca. 50-bp DNA sequences corresponding to the upstream and downstream regions of the *f41A* gene. This linear DNA fragment was then transformed by electroporation into *E. coli* B41, which carried plasmid pKD46, encoding the λ Red recombinase system. The resultant *f41A*:*aph(3′)-IIa* mutant, B41ΔF41, was confirmed by PCR using primer pairs pKD4Fs/B41R and pKD4Rs/B41F. The same method was used to construct a *estA*:*aph(3′)-IIa* mutant, B41ΔSTp, except that primers STpF1 and STpR1 were used to generate *aph(3′)-IIa* flanked by DNA sequences of the upstream and downstream regions of the *estA* gene (also known as *sta1*). Primer pairs STpFseq/pKD4Rs, and pKD4Fs/STpRseq were used to confirm the *estA*:*aph(3′)-IIa* mutation in B41.

Bioluminescent derivatives of *E. coli* B41 and its isogenic F41 and STp knockout mutants were obtained by transforming each strain with the luciferase-encoding plasmid, pXen-13 (Xenogen). The F41 and STp knockout mutant strains (B41ΔF41 and B41ΔSTp, respectively), their bioluminescent derivatives, and the F41-transcomplemented strain, B41ΔF41 (F41), all grew at the same rate *in vitro* as the *E. coli* B41 wild-type.

### Whole Genome Sequencing and Genomic Analyses

Three isolates of *E. coli* B41: wild-type, B41ΔF41 and B41ΔSTp, were sequenced on the NextSeq 500 platform (Illumina) using methods described previously (Ingle et al., 2019). All three isolates passed quality control with phred scores of 33 and average read depth >100. Sequence data for the three isolates were mapped to the publicly available near complete *E. coli* B41 genome (accession AFAH02000000) using snippy v4.3.3^1^. *De novo* assembly was conducted with SPAdes (v3.13.0) (Bankevich et al., 2012). The short read data are available at the European Nucleotide Archive under BioProject PRJEB34804^2^ as: ERR3587299 (B41 wild-type), ERR3587300 (B41ΔSTp), and ERR5014780 (B41ΔF41).

Genomes were screened for known antimicrobial resistance genes with the NCBI database, plasmid replicons with PlasmidFinder database (Carattoli et al., 2014), and for enterotoxin and fimbriae-encoding genes available in the AMRFinderPlus database^3^, performed using ABRicate^4^ with minimum coverage and minimum identity thresholds of 95%.

### Suckling Mouse Assay for ST

*E. coli* strains B41, its isogenic STp and F41 mutants (B41ΔSTp, B41ΔF41), and HS (negative control) were each inoculated into 10 ml CAYE broth and incubated with shaking for 18 h at 37°C. Bacteria were pelleted by centrifugation at 4,000×*g* at 4°C for 20 min, after which 2 ml of each supernatant was removed, and stored on ice until required. STa activity was measured in a suckling mouse assay as described previously with minor modifications (Robins-Browne and Levine, 1981). Briefly, 4–6 days old BALB/c mice were separated from their mothers 2 h before use and then randomly divided into groups of four. Each mouse was inoculated by oral gavage using a 22 G 1.5 inch blunt feeding needle (Cole-Parmer Instruments), with 100 μl of culture supernatant or PBS containing 10 μl of 1% Evans blue dye per ml. Ninety minutes after inoculation, mice were weighed, killed by decapitation, the abdomen was opened and the entire intestinal tract from the proximal duodenum to the distal rectum was removed. The intestines were weighed and the gut to body weight ratio was calculated.

### Assays for F41 Pili

The presence of intact F41 pili on *E. coli* B41 and its isogenic derivates was inferred by the demonstration of the 29 kDa major subunit of F41 using SDS-PAGE, and by the ability of strains to hemagglutinate human blood group A erythrocytes and adhere to HeLa epithelial cell monolayers.

To extract bacterial surface proteins for analysis by SDS-PAGE, *E. coli* B41 and its derivatives were grown overnight in Minca broth without shaking and on Minca agar at 37°C. Cells were harvested by centrifugation of Minca broth and gentle scraping of Minca agar, washed in PBS (pH 7.4), vortexed for 1 min, and incubated at 60°C for 20 min with intermittent vortexing. Samples were then pelleted by centrifugation at 3,000×*g* for 10 min, and the supernatant was transferred to a fresh tube, where it was mixed with NuPAGE lithium dodecyl sulfate sample reducing buffer (Thermo Fisher Scientific) and heated at 70°C for 10 min. Samples (25 μl) were then separated by SDS-PAGE using 12% Bis-Tris NuPAGE gels (Invitrogen), alongside protein ladder standards (BioRAD), and the separated proteins were stained with Coomassie brilliant blue G250. A ∼29 kDa band of interest was excised, trypsin-digested, and analyzed by tandem mass spectrometry at the Proteomics Laboratory, Walter and Eliza Hall Institute of Medical Research, Melbourne, Australia.

For the hemagglutination assays, bacteria were grown overnight in standing cultures of Minca broth and resuspended to a concentration of ∼10^11^ cfu/ml. Doubling dilutions were made and mixed 1:1 with 3% (v/v) human blood group A erythrocytes in 0.9% (w/v) saline with 1% (w/v) methyl-α-D-mannoside (Sigma-Aldrich) on ceramic plates. Agglutination readings were made after gentle rocking at room temperature for 10 min.

Assays for bacterial adherence to cultured HeLa cells were performed as described by Vial et al. (1990) with some modifications. Briefly, bacteria were grown overnight in Minca broth without shaking, and after washing with PBS, approximately 2 × 10^7^ cfu were added to semi-confluent monolayers of HeLa cells on cover slips in 24-well tissue culture plates containing minimal essential media (MEM; ICN) with 8% (w/v) fetal calf serum, 20 mM HEPES (Sigma-Aldrich) and 1% (w/v) methyl-α-D-mannoside. Plates were incubated for 3 h at 37°C in 5% CO_2_, and then washed three times in PBS, fixed in methanol and stained in 10% (w/v) Giemsa stain for 7 min. Coverslips were then washed briefly in Giemsa buffer and allowed to dry before being applied to glass microscope slides with a drop of DePex mounting medium (BHD Merck). After being left to dry overnight, cells were visualized with a Leica DM LB optical microscope. Bacterial strains were designated non-adherent if fewer than 10 of 200 HeLa cells had 5 or more attached bacteria.

### Infection of Neonatal Mice

The virulence of *E. coli* B41 or its derivatives was investigated in genetically verified CBA, BALB/c and C57BL/6 neonatal mice. Bacterial strains used to infect mice were cultured on LA overnight at 37°C. On the day of infection, fresh cultures were collected with a wet sterile cotton swab and suspended in 10 ml of PBS, the turbidity of which was compared to McFarland standards to achieve a dose of ∼2 × 10^6^ cfu in 10 μl. The number of bacteria in the 10 μl inoculum was determined retrospectively by using the surface viable count method of Miles et al. (1938).

Mice were infected perorally by gently pushing the tip of a 10 μl pipette into the side of their mouth, delivering the inoculum in 2–3 μl doses over ∼2 min. Mice were returned to their mothers and monitored for signs of disease, including failure to gain weight, non-responsiveness, dull color, inactivity, shaking, labored breathing, dehydration (skin tenting lasting more than 5 s) and the presence of diarrhea, up to three times daily for 9 days. Disease was classified as “lethal” when mice displayed two or more of these and had to be culled in accordance with our ethics committee’s protocol.

To visualize the location of bacteria in mice, neonatal BALB/c mice in groups of three were infected as described above with *E. coli* B41 or its derivatives transformed with the luciferase-encoding plasmid, pXen-13. After 40 h, mice were killed by decapitation, and the entire intestine from proximal duodenum to distal rectum was excised, teased out on filter paper and visualized semi-quantitatively using an enhanced chemiluminescence system and digital imaging (DNR ChemiBis).

All experiments involving animals were approved by a University of Melbourne Animal Experimentation and Ethics Committee and were performed in accordance with the guidelines for animal experimentation of the Australian National Health and Medical Research Council.

### Statistical Analysis

GraphPad Prism version 8.4.3 was used for plotting graphs and statistical analysis. Quantitative data were analyzed by using one-way ANOVA; qualitative data by using Chi-squared or Fisher’s exact test. Survival curves were derived using Kaplan-Meier estimates, and the log rank test was used to compare different groups. For all tests, a *P*-value of < 0.05 was taken to indicate statistical significance.

## Results

### Susceptibility of CBA, BALB/c, and C57BL/6 Mice to Infection With *E. coli* B41

Duchet-Suchaux et al. (1990) have reported that infant CBA mice are somewhat more susceptible to infection with ETEC strain B41 than BALB/c or C57BL/6 mice. To verify these findings, we infected 1-day old CBA, BALB/c and C57BL/6 mice with 7 × 10^6^ cfu of *E. coli* strain B41. Lethal infections occurred in 4 of 6 (67%) CBA, 15 of 19 (79%) BALB/c and 19 of 20 (95%) C57BL/6 mice (Supplementary Figure 1). Almost 90% of deaths (34 of 38) occurred within 4 days of infection, and no deaths occurred more than 5 days after infection. The difference in mortality of the different mouse strains was not statistically significant (*P* > 0.1, Chi-squared test). All four CBA mice that succumbed to infection, died on day 1 (Supplementary Figure 1), but the difference between the survival curves for the three mouse strains was not significant (*P* = 0.08, log rank test). The flatter survival curve of BALB/c mice (Supplementary Figure 1), suggested that these mice may be best suited to detect subtle differences in virulence between bacterial strains. Accordingly, BALB/c mice were used for all future experiments.

### Genetic Analysis of *E. coli* B41 and Its F41 and STp Knockout Mutants

We undertook whole-genome sequencing of *E. coli* B41 (wild-type) and its F41 and STp knockout mutants to ensure that no significant genetic changes had occurred while constructing the mutants.

All three isolates had >97% coverage of the reference genome. Only one unintended sequence variant was detected between B41ΔF41, and both B41 wild-type and B41ΔSTp. This was a single nucleotide polymorphism (C > A) located in an intergenic region (position 482849 in contig AFAH02000003.1 of reference *E. coli* B41), amongst genes associated with flagellar function. The SNP had no obvious phenotype in terms of growth or motility, or on virulence after trans-complementation with *f41A* (see below). No SNPs were detected between B41 wild-type and B41ΔSTp. As expected, both deletion mutants carried the *aph(3′)-IIa* gene for kanamycin resistance. All three strains had the same plasmid replicon profiles, indicating no loss of plasmids during *in vitro* manipulation. The *f41A* gene (Fidock et al., 1989) was detected in both *E. coli* B41 wild-type and its B41ΔSTp derivative but was absent from B41ΔF41. Similarly, the *estA* gene was detected in B41 wild-type and B4ΔF41 but was absent from B41ΔSTp.

### *In vitro* Characterization of the F41 and ST Deletion Mutants of *E. coli* B41

**FIGURE 1:**
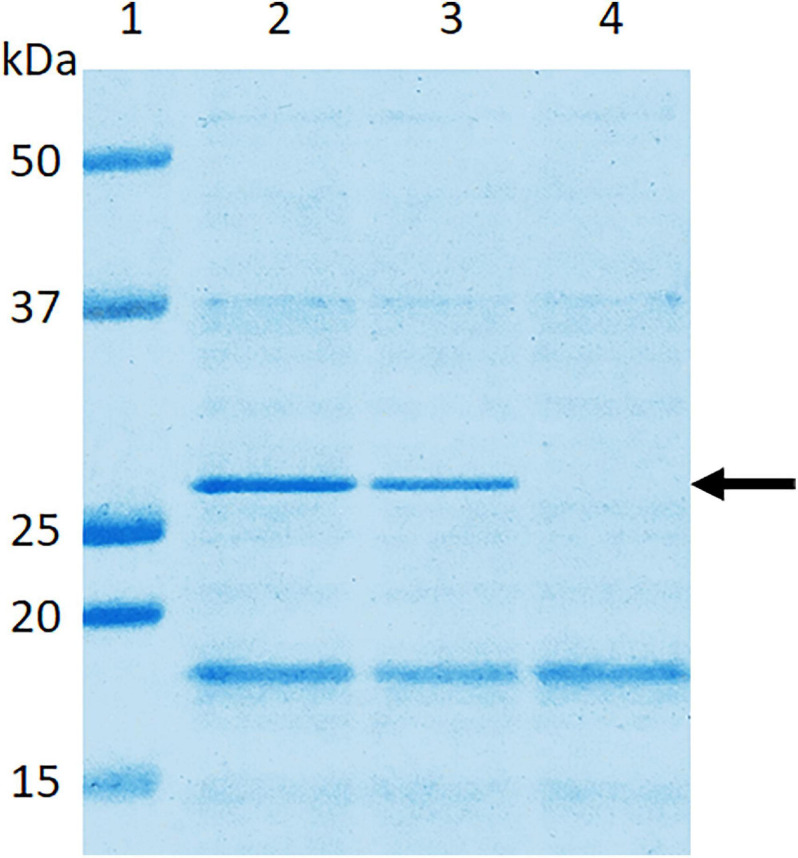
Confirmation in *E. coli* B41ΔF41 of inactivation of the *f41A* gene, which encodes the major structural subunit, F41A, of F41 pili. Surface proteins of bacteria grown overnight in Minca broth and on Minca agar were separated using SDS-PAGE, and stained with Coomassie Brilliant Blue G250. Lane 1, molecular weight standards; lane 2, *E. coli* B41 wild type; lane 3, *E. coli* B41ΔF41 (pACYC184:*f41A*); lane 4, *E. coli* B41ΔF41 (pACYC184). The arrow indicates the ∼29 kDa F41A protein.

**FIGURE 2:**
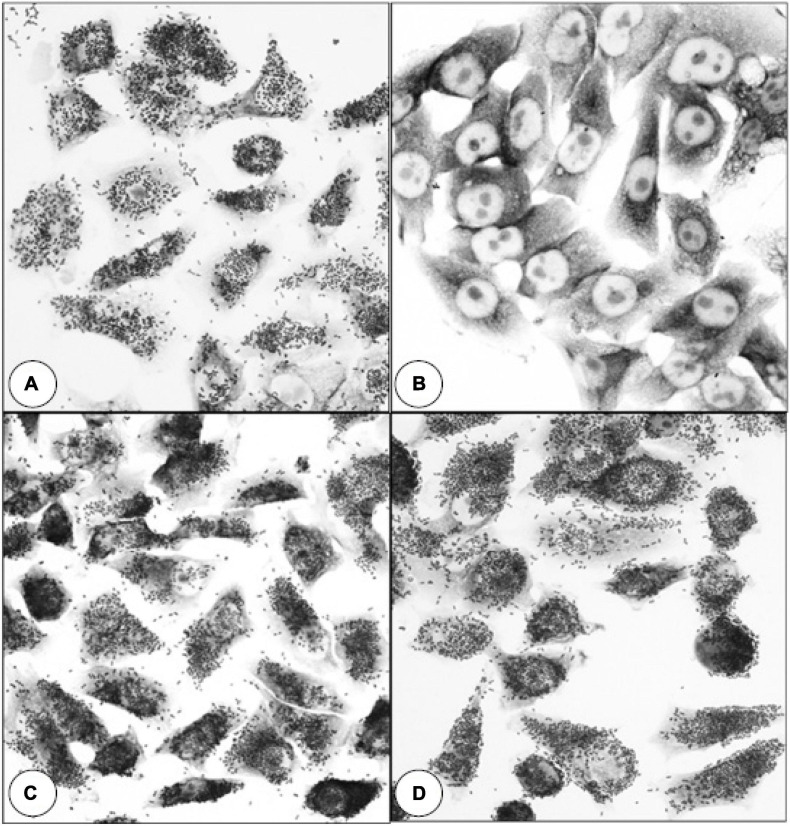
Effect of inactivating *f41A* in *E. coli* B41 on bacterial adherence to HeLa cells. **(A)** *E. coli* B41 wild-type. **(B)** *E. coli* B41ΔF41 (pACYC184). **(C)** *E. coli* B41ΔF41 (pACYC184:*f41A*). **(D)** *E. coli* B41ΔSTp. Similar results were obtained in assays for mannose-resistant agglutination of human blood group A erythrocytes (Supplementary Figure 2).

SDS-PAGE analysis of wild-type *E. coli* B41 showed a distinct band of around 29 kDa that was absent from B41ΔF41 (Figure 1). This band, which was shown by tandem mass spectrometry to be the major structural protein of F41, was restored to B41ΔF41 by trans-complementation of B41ΔF41 with pACYC184:*f41A*. These findings agreed with the results of functional assays for hemagglutination of human erythrocytes, and adhesion to HeLa cells, in which the wild-type and trans-complemented strains were positive, whereas the F41 mutant was negative (Figure 2).

In the suckling mouse assay for STa, both *E. coli* B41 and B41ΔF41 were positive, with mean gut to body weight ratios of 0.093 ± 0.01 (mean ± *SD*) and 0.095 ± 0.01, respectively. In contrast, the values for B41ΔSTp and *E. coli* HS, the negative control, were 0.056 ± 0.002 and 0.058 ± 0.002, respectively. The differences between the two positive strains and B41ΔSTp were significant (*P* < 0.001, one-way ANOVA), whereas the difference between B41ΔSTp and HS was not (*P* > 0.05).

### Investigation of F41 Pili and STp as Virulence Determinants of *E. coli* B41 in Mice

**FIGURE 3:**
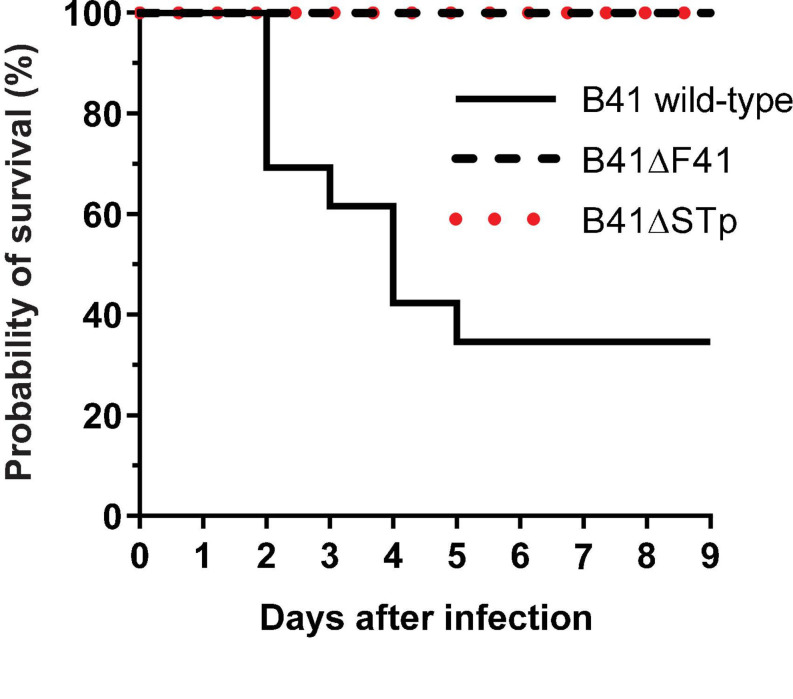
Kaplan-Meier survival analysis of neonatal mice infected perorally with ∼2 × 10^6^ cfu of *E. coli* B41 (*n* = 26) or B41ΔF41 (*n* = 9) or B41ΔSTp (*n* = 14). After inoculation, mice were returned to their mothers and monitored for signs of disease up to three times daily for 9 days. Disease was classified as “lethal” when mice had to be culled due to illness. The probability of survival curves of mice that received either mutant strain differed significantly from those that received the wild-type strain (*P* < 0.0001; log rank test).

None of the nine 1 day-old mice infected with the F41 mutant of *E. coli* B41 succumbed to infection, compared with 17 of 26 (65%) of mice infected with the wild-type strain (*P* = 0.001, Fisher’s exact test). Kaplan-Meier analysis of these data showed that the probability of survival curves of mice infected with the two strains were significantly different from each other (Figure 3; *P* < 0.0001, log rank test).

**FIGURE 4:**
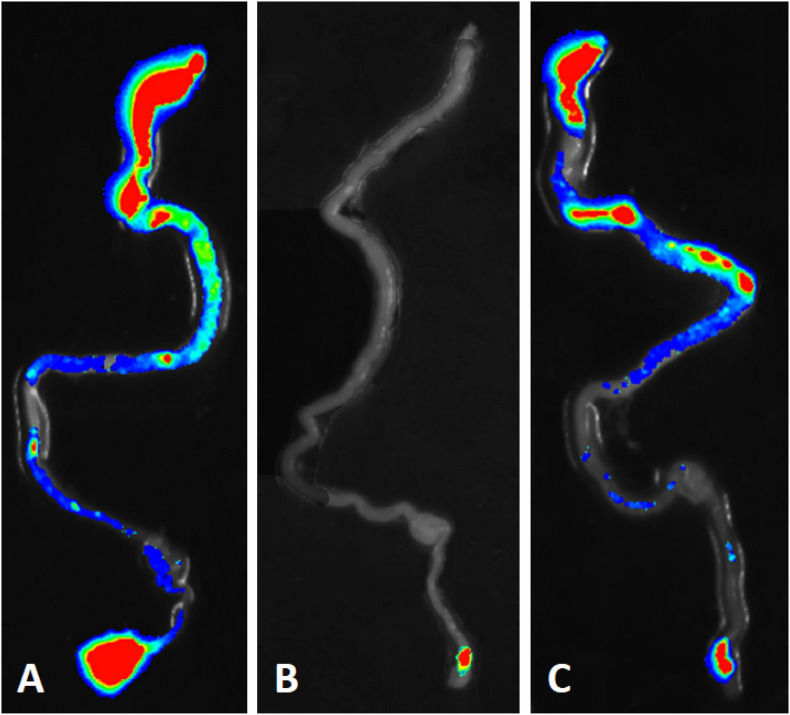
Bioluminescent tracking of *E. coli* B41 (pXen-13) **(A)**, and its F41 **(B)** and STp **(C)** knockout mutants in the intestines of neonatal mice. Neonatal BALB/c mice were infected perorally with derivatives of *E. coli* B41 (pXen-13). After 40 h, mice were killed, their entire intestine was excised and visualized semi-quantitatively using enhanced chemiluminescence and digital imaging. The highest bacterial concentrations are represented by red, and then reduce in number through yellow, green, blue, and finally, no color.

Bioluminescent tracking of *E. coli* B41 (pXen-13) wild-type, and its F41 and STp mutant derivatives 40 h after infection revealed no difference between the wild-type and B41ΔSTp, both of which had colonized various regions of the entire intestine from duodenum to distal rectum, whereas B41ΔF41 was restricted to the distal rectum in all three mice that received this strain (Figure 4). Despite the ability of *E. coli* B41ΔSTp to colonize mouse intestine, none of 14 mice infected with this strain died, compared with 17 of 26 that received the wild type (Figure 3; *P* < 0.0001, Fisher’s exact test).

## Discussion

In a landmark study, Smith and Linggood (1971) showed that both an adhesin (F4/K88) and enterotoxin are required for virulence of ETEC in piglets. Their study was facilitated by the facts that (1) both of the virulence factors they investigated were plasmid encoded, thus making F4- and enterotoxin-negative strains relatively easy to obtain, and (2) piglets are the natural host of F4-positive, enterotoxin-secreting ETEC (Smith and Linggood, 1971). Subsequently, Bertin (1983) used a spontaneous mutant of *E. coli* B41, which did not produce F41, to show that F41 is a virulence determinant of this strain. Given that F41 is chromosomally encoded, a spontaneous mutant may have carried additional mutations that contributed to its loss of virulence. To address issues of this type, Falkow (1988) devised a set of criteria, called Molecular Koch’s postulates, that should be fulfilled before one can be confident that a putative virulence determinant is indeed required by bacteria to cause disease. In essence, these criteria are first, that a strain in which the gene for a putative virulence determinant has been inactivated or deleted is significantly less virulent than the wild-type, and second, that reintroduction of the gene into the attenuated mutant restores virulence. These criteria have not been satisfied for either F41 or STp in ETEC strain B41. Nevertheless, there is considerable circumstantial evidence of the importance of F41 in virulence, including the protective efficacy of vaccines based on F41 (for example Duchet-Suchaux, 1988; Liu et al., 2014), and the passive administration of monoclonal antibodies directed against F41 to mouse dams to protect their suckling neonates from illness caused by *E. coli* B41 (Duchet-Suchaux et al., 1992; van Zijderveld et al., 1998). However, no similar studies have been reported for STp, whose role in the infant mouse model of infection prior to our study was unknown.

In this study, we have shown that both F41 fimbriae and STp are required for virulence of *E. coli* B41 in neonatal mice. Regarding F41, we demonstrated that a genetically well-defined F41 mutant was non-adherent *in vitro* and completely avirulent in mice, and that trans-complementation of this mutant with *f41A*, the F41-encoding gene, restored both hemagglutination and adherence to HeLa cells. Because the trans-complementing plasmid we constructed was unstable in the absence of antibiotic selection, we were unable to investigate the trans-complemented strain in mice, however, whole genome sequencing showed that *E. coli* B41 and B41ΔF41 were identical in every respect except for the *f41A* gene we interrupted to produce the F41 mutant, and one inadvertent nucleotide substitution, which our trans-complementation data indicated was likely to be irrelevant. In addition, by using bioluminescence tracing we established that the F41 mutant colonized the mouse intestine far less effectively than two different F41-bearing strains, namely, *E. coli* B41 wild-type and its isogenic STp deletion mutant, B41ΔSTp.

Regarding STp, we showed for the first time that this toxin is as important as F41 for the virulence of *E. coli* B41 in the mouse model. Because our laboratory is not authorized to clone toxin genes into multicopy plasmids in their natural host, in this case, *E. coli*, we did not attempt to trans-complement B41ΔSTp, but instead performed whole genome sequencing which showed that *E. coli* B41 and B41ΔSTp were identical in every respect except for the gene we interrupted to produce the STp mutant. The ability to analyze whole genome sequences can supplement Molecular Koch’s postulates, because if the data reveal that the mutant is identical to the wild-type in all respects other than the gene being investigated, the need for trans-complementation studies is obviated.

For any animal model of infection to be valid, it should mimic infection in the normal host as closely as possible. With regard to the suckling mouse model of infection with *E. coli* B41, the bacterium’s two essential virulence determinants, F41 and STa, appear to bind to the same receptors and act in mice in the same manner as in *E. coli* B41’s natural hosts. Specifically, STa uses the same receptor, namely, guanylyl cyclase C (GC-C) in mice as in humans and other animals (Swenson et al., 1996; Schulz et al., 1997). Our observation that *E. coli* B41ΔSTp colonized mouse intestine extensively without causing any disease mirrors findings in piglets infected with a spontaneous ST-mutant of an F41-positive ETEC strain (Casey and Moon, 1990). Regarding F41 itself, van Zijderveld et al. (1998) have shown that the same monoclonal antibodies directed against various epitopes of F41 protect both mice and this piglets from infection with F41-positive ETEC. This could be interpreted as indicating that F41 binds to the same or similar receptor(s) in piglets and mice, or that antibodies to different regions of F41 can block its binding regardless of the receptor.

Nevertheless, taken together with the clinical and pathological observations associated with B41 infection of neonatal mice published previously (Duchet-Suchaux, 1980; Bertin, 1983; Newsome et al., 1987), our demonstration that both F41 and STp are required for full virulence of *E. coli* B41 in neonatal mice, validates this model completely. Our findings also indicate that the model may be useful for screening vaccine candidates for ST-producing ETEC by using active immunization of dams followed by experimental challenge of suckling pups as described elsewhere (Duchet-Suchaux, 1983; Liu et al., 2014).

## Data Availability Statement

The datasets presented in this study can be found in online repositories. The names of the repository/repositories and accession number(s) can be found below: https://www.ebi.ac.uk/ena, ERR3587299; ERR3587300; ERR5014780.

## Ethics Statement

The animal study was reviewed and approved by the University of Melbourne Small Laboratory Animal Ethics Committee.

## Author Contributions

CC, DH, JP, MT, and RR-B designed and performed most of the experiments and contributed to the analysis and interpretation of data. DH, KIA, JP, VB-W, and KA carried out some experiments. DI and SB analyzed the genome sequences. CC, DH, KA, and RR-B prepared the figures for publication. CC and RR-B wrote the manuscript. All authors reviewed the manuscript before submission.

## Conflict of Interest

The authors declare that the research was conducted in the absence of any commercial or financial relationships that could be construed as a potential conflict of interest.

## Footnotes

1 https://github.com/tseemann/snippy

2 https://www.ebi.ac.uk/ena/browser/view/PRJEB34804

3 https://www.ncbi.nlm.nih.gov/pathogens/antimicrobial-resistance/AMRFinder/

4 https://github.com/tseemann/abricate
