# Supplementary material for: Re-evaluation of a Neonatal Mouse Model of Infection With Enterotoxigenic Escherichia coli
Source: Front Microbiol. 2021 Mar 18;12:651488. doi: 10.3389/fmicb.2021.651488 (PMC8013722; doi:10.3389/fmicb.2021.651488)
Supplement: Supplementary file 1 [file Data_Sheet_1.PDF]

# Supplementary Materials

**Supplementary Table 1.** PCR primers used in this study.

**Supplementary Figure 1.** Kaplan-Meier survival analysis of neonatal CBA (n = 6), C57BL/6 (n = 20) and BALB/c (n = 19) mice infected perorally with ~2 x $10^6$ cfu of *E. coli* B41. After inoculation, mice were returned to their mothers and monitored for signs of disease up to three times daily for 9 days. Disease was classified as 'lethal' when mice had to be culled due to illness. The probability of survival curves of the different mouse strains were not significantly different from one another (P >0.05; log rank test).

**Supplementary Figure 2**. Effect of inactivating *f41A* in *E. coli* B41 on bacterial-mediated, mannose-resistant hemagglutination of human group A red blood cells. A, *E. coli* B41 wild-type; B, *E. coli* B41ΔF41(pACYC184); C, *E. coli* B41ΔF41(pACYC184::*f41A*), and D, *E. coli* HS.

**Supplementary Table 1.** PCR primers used in this study

| Primer | Used to: | Sequence (5'-3') |
|---|---|---|
| hstA-F | Amplify *estA* | TTTCCCCTCTTTTAGTCAGTCAA |
| hstU-R | Amplify *estA* | GCAGGATTACAACAAAGTTCACAGCAG |
| F41F | Amplify *f41A* | CAGGGACTTTCATCTTTTAG |
| F41R | Amplify *f41A* | AGTCCATTCCATTTATAGGC |
| STpFl | Generate *estA::aph(3')-IIa* knockout fragment[a] | CTGTATTATCTTTCCCCTCTTTTAGTCAGTCAACTGAATCACTTGACTC**TGTGTAGGCTGG AGCTGCTTC** |
| STpRl | Generate *estA::aph(3')-IIa* knockout fragment[a] | CAGCACAGGCAGGATTACAACAAAGTTCACAGCAGTAAAATGTGTTGTTCG**GTCCATATGAATATCCTCCTT AGTTCC** |
| STpFseq | Amplify *aph(3')-IIa* insert in *estA* | CCTCGACATATAACATGATGCAAC |
| STpRseq | Amplify *aph(3')-IIa* insert in *estA* | TAGAGGGAATCAAAATAAAGATTCC |
| pKD4Fs | Amplify *aph(3')-IIa* insert in *estA* and *f41A* | TGACGAGTTCTTCTGAGCGGGAC |
| pKD4Rs | Amplify *aph(3')-IIa* insert in *estA* and *f41A* | TCTAGCTATCGCCATGTAAGCC |
| F41.Fl | Generate *f41A::aph(3')-IIa* knockout[b] | GGTGATTATTTTATGAAAAAGACTCTGATTGCACTGGCTGTGGCTGCATCA**TGTGTAGGCTGGAGCTGCTTC** |
| F41.Rl | Generate *f41A::aph(3')-IIa* knockout[b] | GGAAGGGCAGAAAGATGATTAACTATAAATAACGGTGATAGTCACTGGAGC**GGTCCATATGAATATCCTCC TTAGTTCC** |
| F41.Fseq | Amplify *aph(3')-IIa* insert in *f41A* | TGCAGGTGGAAGACTGAGGATTC |
| F41.Rseq | Amplify *aph(3')-IIa* insert in *f41A* | GAATATCTGCATGTGGCGCTGC |
| M13F[c] | Amplify *f41A* from TOPO | GTAAAACGACGGCCAG |
| M13R[c] | Amplify *f41A* from TOPO | CAGGAAACAGCTATGAC |

[a] Underlined sequence is complementary to *estA;* boldface sequence is complementary to pKD4.
[b] Underlined sequence is complementary to *f41A, the* F41 major subunit gene; boldface sequence is complementary to pKD4.
[c] Included in the TOPO cloning kit (Invitrogen)

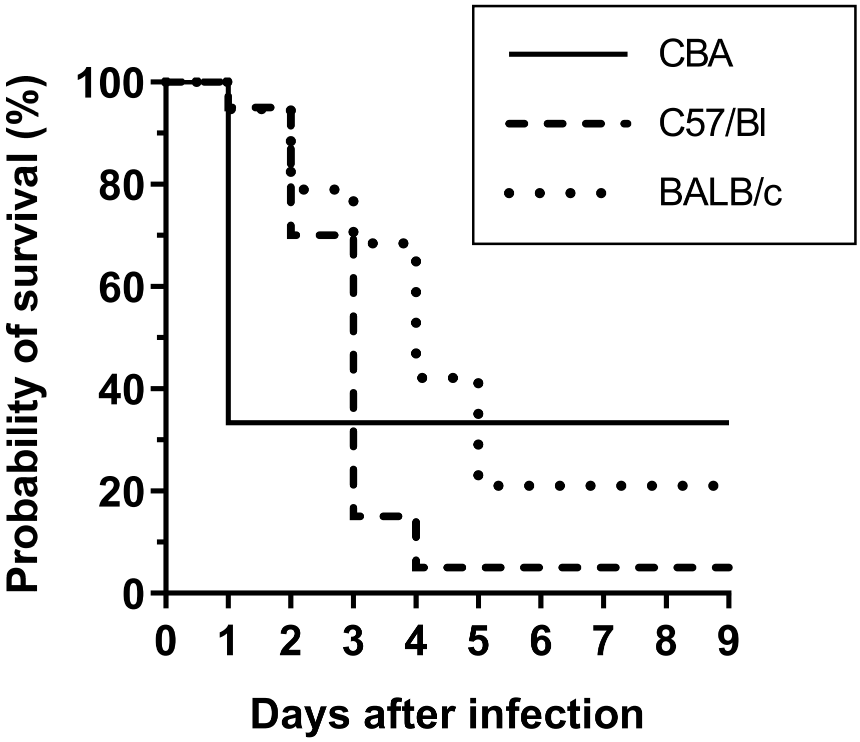


**Supplementary Figure 1:** Kaplan-Meier survival analysis of neonatal CBA (n = 6), C57BL/6 (n = 20) and BALB/c (n=19) mice infected perorally with ~2 x $10^6$ cfu of *E. coli* B41. After inoculation, mice were returned to their mothers and monitored for signs of disease up to three times daily for 9 days. Disease was classified as 'lethal' when mice had to be culled due to illness. The probability of survival curves of the different mouse strains were not significantly different from one another (P >0.05; log rank test).

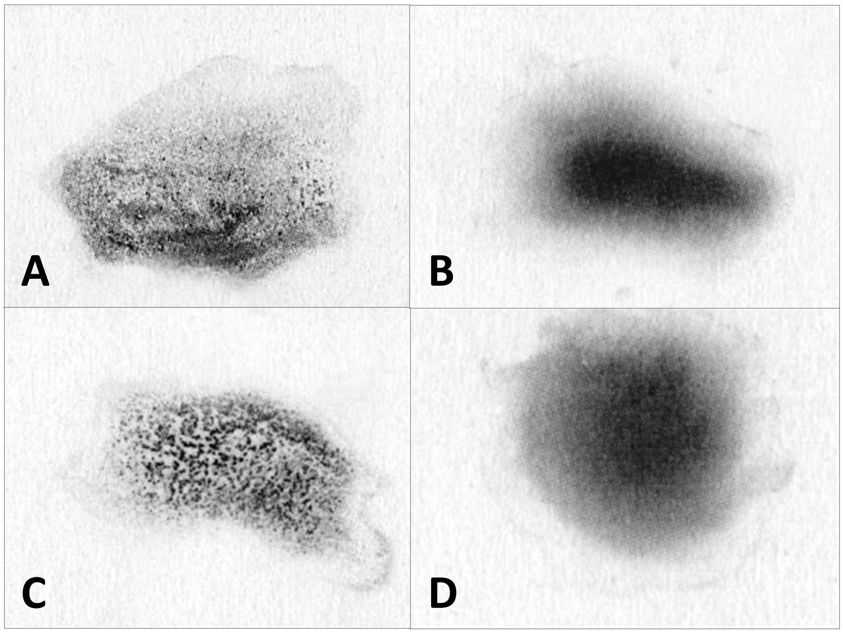


**Supplementary Figure 2**. Effect of inactivating *f41A* in *E. coli* B41 on bacterial-mediated, mannose-resistant hemagglutination of human group A red blood cells. A, *E. coli* B41 wild-type; B, *E. coli* B41ΔF41(pACYC184); C, *E. coli* B41ΔF41(pACYC184::*f41A*), and D, *E. coli* HS.
